# Gene–environment interaction: New insights into perceived parenting and social anxiety among adolescents

**DOI:** 10.1192/j.eurpsy.2020.62

**Published:** 2020-06-08

**Authors:** Viktoria Chubar, Karla Van Leeuwen, Patricia Bijttebier, Evelien Van Assche, Guy Bosmans, Wim Van den Noortgate, Ruud van Winkel, Luc Goossens, Stephan Claes

**Affiliations:** 1 Mind-Body Research Group, Department of Neuroscience, KU Leuven, Leuven, Belgium; 2 Parenting and Special Education Research Unit, Faculty of Psychology and Educational Sciences, KU Leuven, Leuven, Belgium; 3 School Psychology and Development in Context, Faculty of Psychology and Educational Sciences, KU Leuven, Leuven, Belgium; 4 University Psychiatric Center KU Leuven, Leuven, Belgium; 5 Clinical Psychology, Faculty of Psychology and Educational Sciences, KU Leuven, Leuven, Belgium; 6 Department of Methodology of Educational Sciences, Faculty of Psychology and Educational Sciences, KU Leuven, Leuven, Belgium; 7 Center for Contextual Psychiatry, Department of Neuroscience, KU Leuven, Leuven, Belgium; 8 School Psychology and Child and Adolescent Development Research Unit, Faculty of Psychology and Educational Sciences, KU Leuven, Leuven, Belgium

**Keywords:** Adolescence, gene–environment interactions, parenting, social anxiety

## Abstract

**Background.:**

Social anxiety symptoms (SAS) are among the most common mental health problems during adolescence, and it has been shown that parenting influences the adolescent’s level of social anxiety. In addition, it is now widely assumed that most mental health problems, including social anxiety, originate from a complex interplay between genes and environment. However, to date, gene–environment (G × E) interactions studies in the field of social anxiety remain limited. In this study, we have examined how 274 genes involved in different neurotransmission pathways interact with five aspects of perceived parenting as environmental exposure (i.e., support, proactive control, psychological control, punitive control, and harsh punitive control) to affect SAS during adolescence.

**Methods.:**

We have applied an analytical technique that allows studying genetic information at the gene level, by aggregating data from multiple single-nucleotide-polymorphisms within the same gene and by taking into account the linkage disequilibrium structure of the gene. All participants were part of the STRATEGIES cohort of 948 Flemish adolescents (mean age = 13.7), a population-based study on the development of problem behaviors in adolescence. Relevant genes were preselected based on prior findings and neurotransmitter-related functional protein networks.

**Results.:**

The results suggest that genes involved in glutamate (*SLC1A1*), glutathione neurotransmission (*GSTZ1*), and oxidative stress (*CALCRL*), in association with harsh punitive parenting, may contribute to social anxiety in adolescence. Isolated polymorphisms in these genes have been related to anxiety and related disorders in earlier work.Conclusions: Taken together, these findings provide new insights into possible biological pathways and environmental risk factors involved in the etiology of social anxiety symptoms’ development.

**Conclusions.:**

Taken together, these findings provide new insights into possible biological pathways and environmental risk factors involved in the etiology of social anxiety symptoms’ development.

## Introduction

As is the case in the majority of psychiatric disorders as well as other complex behavioral traits, the etiology of social anxiety disorder (SAD) can be attributed to the interplay between genetic and environmental risk factors [1]. SAD typically starts with social anxiety symptoms (SAS) during late-childhood and mid-adolescence, a period characterized by an increase in environmental complexity. Once present, SAS can lead to a variety of maladjustments such as poor social functioning, an overall reduction in life quality, and physical health complications [2–4]. Furthermore, it has been shown that anxiety symptoms occurring in childhood and adolescence have a strong persistence over time [5–7] and are associated with a poorer treatment outcome [8,9]. This highlights the need for early SAS identification [10]. Therefore, new insights into the development and risk factors of SAS are of great importance.

Previous research has shown that parenting practices can be divided into dimensions that are differentially linked to behavior acting as a promotive or risk factor for development of mental health problems including anxiety in adolescents [11,12]. It was also shown that an adverse family environment and parenting are associated with the etiology of SAS [13,14]. Specifically, abusive and overcontrolling parental behavior are linked to higher levels of SAS both in a sample from the general population and in clinical SAD outpatients [15–17]. These negative parenting practices have been hypothesized to reduce the child’s opportunity to learn the skills required for good socialization and thereby increase anxiety symptoms and social withdrawal. Furthermore, exposure to such parent-driven chronic adversities have a negative impact on the stress response system’s functioning, resulting in sensitization to stressors that facilitate SAS [18,19]. Therefore, parenting and the family environment play an important role in the development of SAS among youth.

Another important etiological aspect of SAS development is rooted in genetics. A variety of twin studies have shown that SAS has a heritable basis [1,20]. SAD heritability rates are estimated to be between 13 and 76% (for a detailed review, please see Moreno et al. [17]). However, the variance associated with both genetic and environmental factors varies widely between studies [16,17]. This variability seems to originate from the high heterogeneity of twin cohorts. For example, the variance associated with genetic factors has been found higher in children and adolescents compared to adults [1,16]. These results suggest that genetic and environmental factors might have a different impact throughout development, stressing the importance of investigating their influence over time. However, to date, the literature aimed at improving our understanding of the genetic basis of SAS and SAD remains limited.

The first insights into the genetic architecture of SAD vulnerability came from candidate gene (CG) studies and two genome-wide association studies (GWASs). In the study of Trzaskowsk et al. [21] on anxiety traits in 2,810 seven-year-old children, no single nucleotide polymorphism (SNP) reached the genome wide threshold for significance, and the SNPs that showed the lowest *p* values with relation to social anxiety traits (rs2772129 and rs2922037) could not be replicated in another similar cohort of 4,804 children. More promising were the findings of an even larger GWAS on anxiety traits by Stein et al. [18], who reported two SNPs that were significantly associated with social anxiety (rs78924501 on Chr 1 in African American and rs708012 on Chr 6 in European American samples). Still, the data from these studies explain only a very small proportion of SAS’s heritability.

One of the reasons for the relative lack of success of GWAS may be related to the lack of sufficient power when applying standard corrections for multiple testing. In addition, the above mentioned GWAS only investigated main effects and did not take into account environmental risk factors. This can lead to an oversimplification of the multidimensional etiology of such complex symptoms. As a result, some SNPs that are in fact associated with the trait might not be identified [22,23]. However, such SNPs might be detected once relevant environmental factors and additional effects of age and gender are taken into consideration, which can be achieved through a gene—environment interaction (G × E) approach [24]. Contrary to CG and GWAS, the main focus of G × E studies is on the interaction effects between genetic variants and environmental factors. Within this framework, it is assumed that the risk of developing the disease is increased when people with susceptible genotypes are exposed to adverse environmental conditions [25]. In addition, factors such as age and gender are typically also taken into account. However, to date, G × E studies in the field of social anxiety have investigated only a very limited number of genes, mainly within the oxytocin and serotonin neurotransmission systems [26–28].

In this study, we investigate how 274 genes involved in nine neurotransmission systems (serotonin, dopamine, hypothalamic pituitary adrenal [HPA] axis, oxytocin, GABA, glutamate, choline, noradrenergic, and the clock pathway) and perceived parenting behaviors (as environmental exposures) interact in explaining SAS during adolescence. Such hypothesis-driven preselection of biologically relevant genes allows us to incorporate biological knowledge into G × E analyses and helps to increase statistical power (by reducing the effect of multiple test corrections), which is important for relatively smaller sample sizes [29]. Genes were selected based on their involvement in neurotransmitter-related functional protein networks. It was shown that highly complex behaviors can arise from a restricted set of gene families, by a tightly regulated interaction network of proteins encoded by these genes [30]. The functional networks are constructed based on the proteins’ direct physical and their indirect functional interactions, such as catalysis of subsequent reactions in a metabolic pathway and ability to regulate each other transcriptionally or posttranscriptionally [30,31]. To study these G × E interactions, we have used a statistical approach based on Brown’s method [32,33], which allows preserving power by taking genes, rather than SNPs, as the main unit of analysis.

## Methods

### Participants

All participants were part of the STRATEGIES cohort (i.e., Studying Transactions in Adolescence: Testing Genes in Interaction with Environments), a population-based study on the development of problem behaviors in adolescence. After active written informed consent was provided, participants were asked to fill out questionnaires and to provide a saliva sample for further genotyping. The STRATEGIES cohort consists of 1,111 adolescents with genetic information available for 1,103 participants. In this study, only participants of Caucasian origin and with available data on social anxiety and perceived parenting were included. In addition, if two siblings participated, only one child per family was randomly selected, which resulted in a final sample of 948 adolescents (mean age = 13.78 [standard deviation, SD = 0.92]; 50.5% boys).

### Social anxiety and perceived parenting

SAS were assessed using the self-reported Dutch version of the Social Anxiety Scale for Adolescents (SAS-A) [34], a 12-item short version of the SAS-A [35]. A mean score was calculated, with higher scores indicating greater levels of SAS.

Perceived parenting, as reported by adolescents, was measured via the Leuven Adolescent Perceived Parenting Scale [36] and Parental Behavior Scale–Short [37]. All items were rated by adolescents on a 5-point scale ranging from 1 = (almost) never to 5 = (almost) always. Based on the abovementioned parenting scales, we computed five parenting dimensions (support, proactive control, psychological control, punitive control, and harsh punitive [HP] control) by grouping items and calculating mean scores as described in Janssens et al. [38]. These parenting dimensions represent particular features of parenting that are hypothesized to have an effect on developmental outcomes and child behavioral adjustment. In all subsequent analyses, these five parenting dimensions were used as indicators of the quality of the adolescents’ family environment. Descriptive statistics of the SAS-A and parenting dimensions are available in Table1. For a more comprehensive description regarding the STRATEGIES cohort, data collection, and perceived parenting measurements, we refer to previous publications by our group [38,39].

### Genetic information

In total, 5,052 SNPs in 344 genes involved in nine neurotransmitter pathways (serotonin, dopamine, the HPA-axis, oxytocin, GABA, glutamate, choline, noradrenergic neurotransmission, and the clock pathway) were genotyped.

The genotyping quality control was done based on the protocols of Anderson et al. [40] and Purcell et al. [41]. In brief, SNPs with a call rate of less than 98% and/or a minor allele frequency of less than 1% were excluded. The population structure was checked and confirmed to be homogeneous via a principal component analysis, and all SNPs were in Hardy–Weinberg equilibrium. In the next step, the linkage disequilibrium (LD) matrices were calculated for all available SNPs (using PLINK v1.07). A more detailed description of the LD matrices, selected genes, and SNPs, and the applied quality control methods are available in our previous publication [32].

### Statistical analysis

In brief, we first applied a linear regression model, in which we tested the interaction effects of each SNP (G) with each parenting dimension (E). Regression models were fitted for each of five parenting dimensions separately. As severe degrees of HP control were relatively rare in our data set—which resulted in a strong right skewness of this variable—we applied a sensitivity analysis, in which we distinguished between no presence of HP control and any presence.

All regression analyses were adjusted for the potentially confounding effects of gender and age, based on a recommendation by Keller [42]. The output of the regression models (*p* value of G × E interactions) was taken for further analysis. Next, all SNPs in the output file were linked to their corresponding genes.

Since single SNPs within a specific gene are often in LD, the *p* values obtained from the interaction of these SNPs with environment are not independent either. To account for this, we applied a gene-based analysis using Brown’s method with adjustment for LD [33]; we used poolr, a recently developed R package for pooling the results from (dependent) tests. The detailed description of Brown’s method for G × E analysis can be found in existing literature [32]. The gene-based analysis with Brown’s method allows us to calculate a unified *p* value per gene, which is adjusted for nonrandom association of SNPs, particularly the LD structure of the gene which is estimated based on the LD matrices. Only genes with two or more SNPs were selected for further analysis, which resulted in 274 genes. Bonferroni correction was applied based on the number of genes (adjusted *p* value = 1.8 × 10^−4^). When significant G × E interactions were found, a permutation test with 100,000 permutations was used to verify the validity of our findings [43]. In addition to that, we calculated a genomic control (GC) coefficient to check for bias in the distribution of the test statistic [44]. The analysis was done using R [45].

## Results

**Figure 1.**
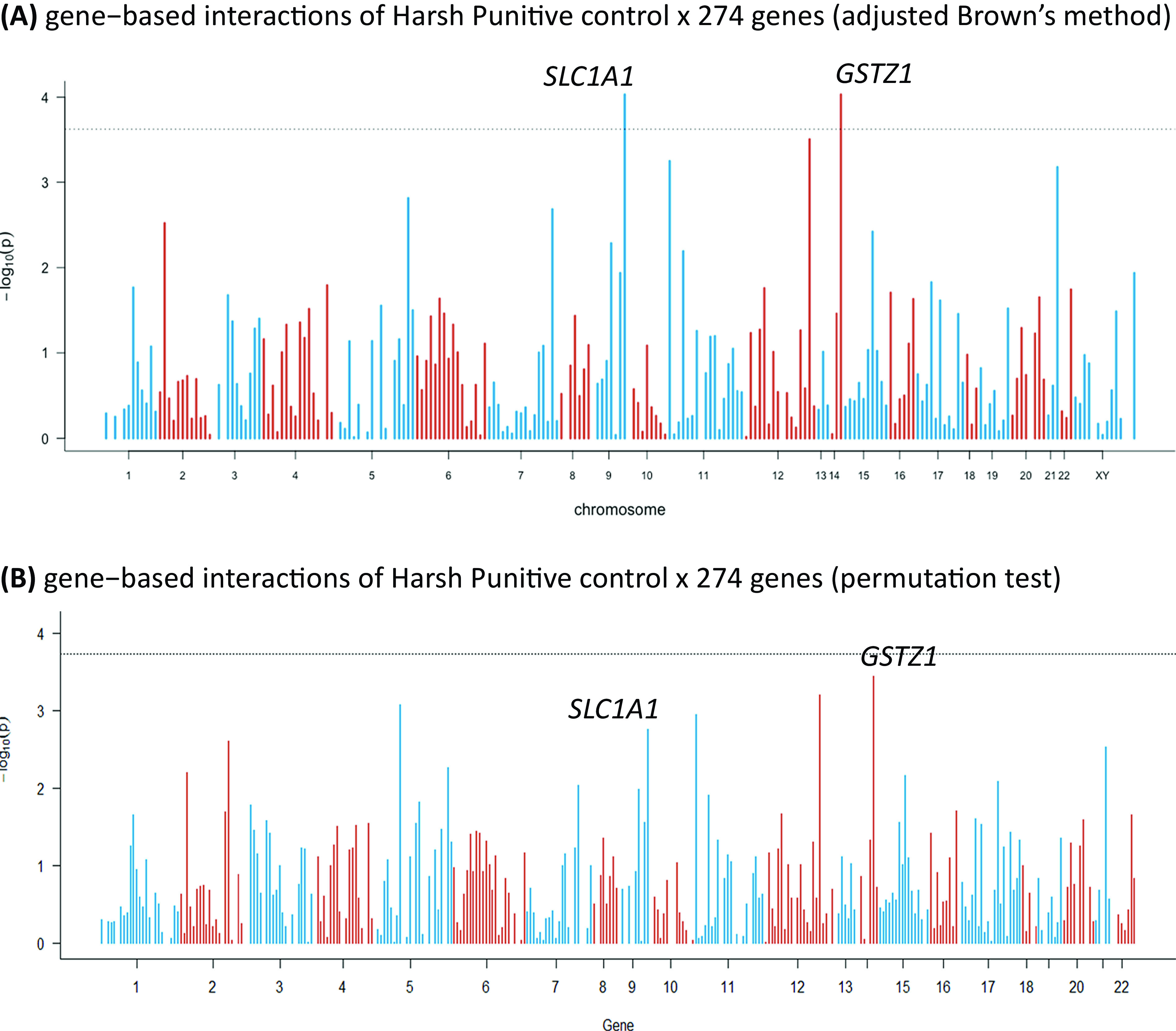
Manhattan plots of gene-based interactions of Harsh Punitive Control × 274 genes: (A) results of adjusted Brown’s method and (B) results of permutation test. Bonferroni correction: 1.8 × 10^−4^; each bar represents a gene. The dashed line represents the significance level after the Bonferroni correction.

Significant gene-based interactions were present for one parenting dimension, that is, “HP control,” in interaction with two genes (the neuronal glutamate transporter excitatory amino acid carrier 1 (*SLC1A1*), *p* = 9.3 × 10^−5^, and the glutathione transferase zeta 1 (*GSTZ1*), *p* = 9.3 × 10^−5^; Figure 1A). Interactions with other parenting dimensions were not significant. More information regarding results for all parenting dimensions are available in the Supplementary Materials.

The GC showed strong inflation, with an inflation factor of *λ* = 1.5. The results of the permutation test for the *GSTZ1* gene came close to significance, but without actually meeting the threshold (Figure 1B).

**Table 1.** Descriptive statistics of the sample and the main variables: SAS-A and the five parenting dimensions obtained via the Leuven Adolescent Perceived Parenting Scale and the Parental Behavior Scale–Short.

Variables	Minimum	Maximum	Mean	SD	Cronbach’s alpha
Age	11.3	17.02	13.7	0.92	–
SAS-A	1	5	2.4	0.78	0.92
Support	1.4	5	3.9	0.64	0.94
Proactive control	1.8	5	3.7	0.62	0.84
Psychological control	1	4.4	1.9	0.63	0.90
Punitive control	1	5	2.2	0.99	0.88
Harsh punitive control	1	4.8	1.2	0.52	0.89

Abbreviations: SAS-A, Social Anxiety Scale for Adolescents; SD, standard deviation.

**Figure 2.**
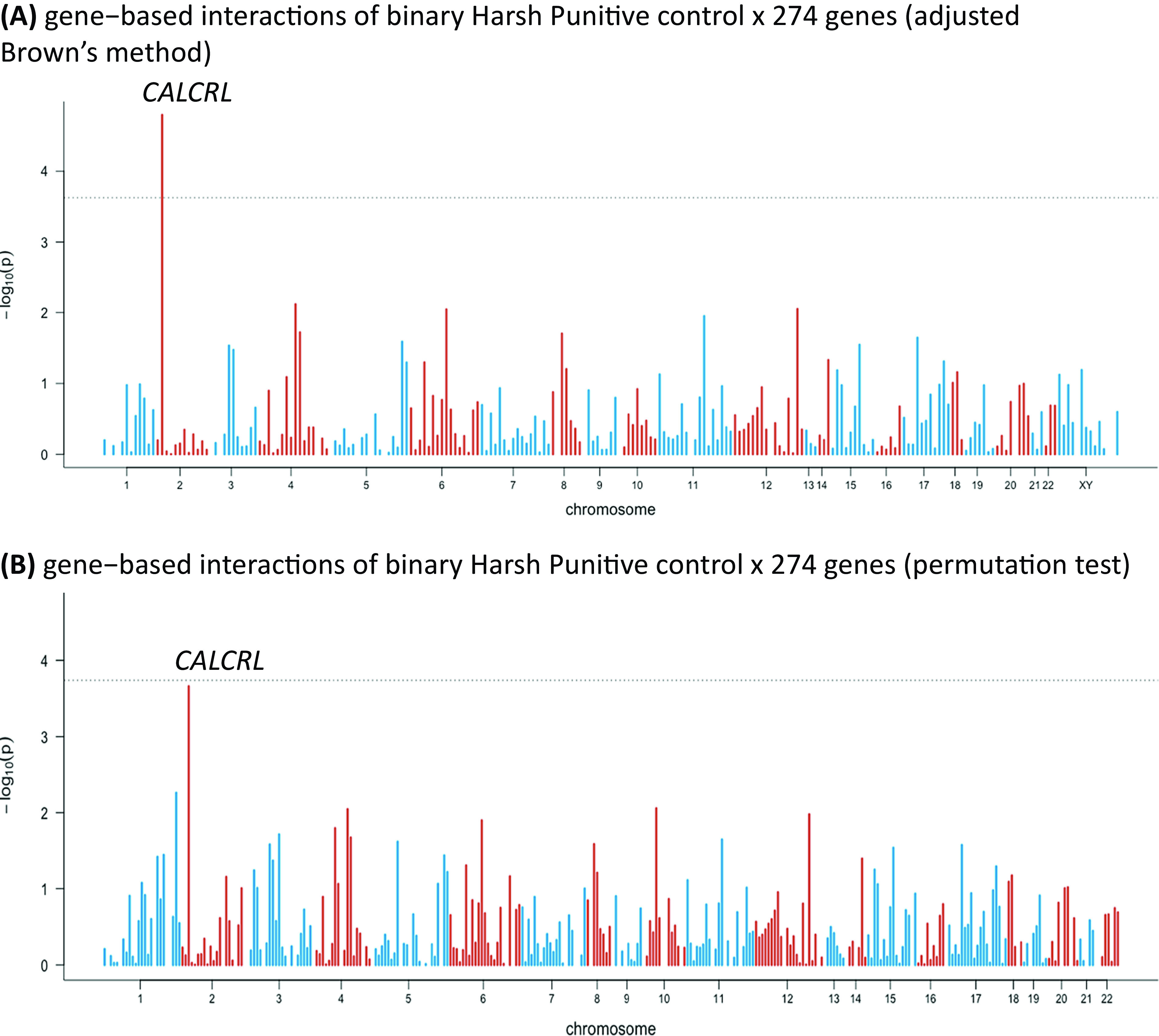
Manhattan plots of gene-based interactions of binary Harsh Punitive Control × 274 genes: (A) results of adjusted Brown’s method and (B) results of permutation test. Bonferroni correction: 1.8 × 10^−4^; each bar represents a gene. The dashed line represents the significance level after the Bonferroni correction.

In the sensitivity analysis, we distinguished between no presence and any presence of HP control and repeated the analysis. We used median split to dichotomize HP control. One gene showed a significant interaction with HP control (the calcitonin receptor-like gene [*CALCRL*]; *p* value = 1.59 × 10^−5^; inflation factor *λ* = 1.05; Figure 2A). The permutation test came close to significance, but without reaching the threshold (Figure 2B). Interestingly, in the first analysis, the *CALCRL* gene was also close to significance (see Supplementary Materials).

## Discussion

Using the data from the STRATEGIES cohort of almost 1,000 Flemish adolescents, we have examined how genes and perceived parenting behavior as environmental exposure interact in association with SAS during adolescence. To study these gene–environment interactions, we have applied a gene-based analysis using Brown’s method with adjustment for LD that allows studying genetic information at the gene level by aggregating data from multiple SNPs within the same gene. This approach can help to uncover genes and biological pathways that interact with the environment during development. In our analysis, we included SNPs that belong to 274 genes involved in nine neurotransmission systems: serotonin, dopamine, HPA-axis, oxytocin, GABA, glutamate, choline, noradrenergic, and the clock pathway.

We found significant gene–environment interactions for only one parenting dimension (HP control). Two genes remained significant after we controlled for multiple testing: *SLC1A1* and *GSTZ1.* However, the genomic inflation factor was high (*λ* = 1.5). The results of the permutation test, used to verify the accuracy of our interactions, showed that *SLC1A1* or *GSTZ* were among the top significant genes, but neither reached significance after adjusting for Bonferroni correction (Figure 1B). The data were checked for population structure and familial relationships, and these were therefore excluded as possible reasons for increased genomic inflation. Given the skewness of the HP control variable, a bigger sample size might be needed for studies of this parenting dimension in the general population. G × E studies of HP control such as this one are very relevant given that the degree of reported HP control is strongly associated with maltreatment, which is a well-known risk factor for mental health problems, including social anxiety [46,47].

Next, we used a sensitivity analysis to distinguish between the absence and presence of HP control. One gene came out significant: *CALCRL.* This gene was also among the most strongly associated genes in the first analysis (for details, please see Supplementary Materials). The result of the permutation test was close to significance, without reaching the expected threshold (Figure 2B).

Studies on *SLC1A1*, *GSTZ1, CALCRL*, and psychopathology in humans are scarce. However, the available literature suggests that these genes may be involved in the development of anxiety.

*SLC1A1* is a glutamate transporter gene that is highly expressed in the brain. Polymorphisms in *SLC1A1* have been associated with early onset obsessive–compulsive disorder in males [48,49] and in a general sample [50]. Some SNP variants in *SLC1A1* are also reported to significantly increase the likelihood of post-traumatic stress disorder in combat-exposed veterans [51]. In addition, polymorphisms in *SLC1A1* are associated with the severity of anxiety symptom in children with autism spectrum disorder [52].

*GSTZ1* is a multifunctional enzyme that belongs to the glutathione S-transferase enzymes superfamily. It is primarily responsible for glutathione dependent metabolism including the conjugation of glutathione to substrates and is involved in oxidative stress regulation [53,54]. An association was shown between variants in *GSTZ1* and an increased susceptibility to early onset of bipolar disorder [55].

Interestingly, changes in glutamate levels and increases in cellular oxidative stress were reported in animal models of social isolation rearing that are commonly compared with psychological stressors in humans [56]. In addition, changes in expression levels of genes involved in oxidative stress, in particular, in those genes related to glutathione enzymes, were also linked to anxiety-related phenotypes [57]. In human studies, cortical glutamate levels have been associated with increased anxiety [58], and level changes in cortical glutamate have also been reported in pharmacologically induced anxiety [59]. Together, the abovementioned studies indicate that biological pathways that regulate oxidative stress and glutamate neurotransmission are related to anxiety-like behavior and can be sensitive to stressful life events during development.

Although our results should be interpreted with caution, our findings align with previous studies and suggest that polymorphisms in genes involved in glutamate neurotransmission (*SLC1A1*) and in oxidative stress (*GSTZ1*), together with harsh parenting might facilitate increases in SAS. It is worth knowing, however, that biological pathways associated with *SLC1A1* and *GSTZ1* seem to be related to anxiety symptoms in general, rather than to social anxiety specifically. This may be plausible, as the same biological vulnerabilities could underlie both social and general anxiety disorders.

*CALCRL* plays an important role in neovascularization control [60] and is involved in inflammation, blood pressure regulation, vascular biology, and cell proliferation [60–62]. Recently, it was suggested as a potential target for migraine treatment [63]. Increased inflammation is reported in relation to anxiety [64], but also in the majority of other stress-related disorders [65], as exposure to repeated and chronic stressful events was repeatedly associated with increase in pro-inflammatory processes [65,66].

Our results from the sensitivity analysis may suggest that binary environmental variables might make the model less specific to certain type of environmental exposure because more specific information available in the environmental variable might have been lost. In this way, HP control, which includes harsh punishment parental practices (such as physical punishment), after coded as being present or not might represent a more general exposure to stressful life experiences or adversity, rather than specific child–parent interactions. This can explain why in the sensitivity analysis, *CALCRL*, a gene involved in inflammation, a biological process linked with stress, became more prominent.

Therefore, this study suggests that interactions between genes and social environment play an important role in the etiology of anxiety-related disorders. However, it is important to note that more studies are needed to investigate further the interaction of genes involved in glutamate, glutathione neurotransmission, and oxidative stress in association with social environmental factors and the development of social anxiety-related symptoms.

Our study also shows that a hypothesis-driven preselection of genes, based on their functional protein networks, allows researchers to incorporate biological knowledge into G × E analyses. Such an approach allows researchers to focus on biological pathways that might be affected by adverse environments during sensitive developmental time periods. This allows a better understanding of the contribution of both genes and environment along the developmental time span. Our results also show the importance of applying a control for genomic inflation and permutation tests in G × E interaction studies.

Several limitations should be acknowledged. First of all, this study is based on cross-sectional data; therefore, it is difficult to establish a temporal relationship between variables. Secondly, both SAS and parenting were measured via self-reported questionnaires, which potentially introduces bias [67]. This study also did not include a replication sample, as for reliable replication we would have needed a sample with similar age, outcome, and environmental variables, and currently genetically informed studies within the field of developmental psychology are scarce.

## Data Availability

The dataset used in this study is not publicly available, because of the presence of sensitive information. However, the data are available from the last author, upon reasonable request and arrangement of data sharing agreements.

## Supplementary material

For supplementary material accompanying this paper visit http://dx.doi.org/10.1192/j.eurpsy.2020.62. click here to view supplementary material

## References

[1] Scaini S, Belotti R, Ogliari A. Genetic and environmental contributions to social anxiety across different ages: a meta-analytic approach to twin data. J Anxiety Disord. 2014;28(7):650–656. 25118017 10.1016/j.janxdis.2014.07.002

[2] Ooi LL, Nocita G, Coplan RJ, Zhu J, Rose-Krasnor L. Beyond bashful: examining links between social anxiety and young children’s socio-emotional and school adjustment. Early Child Res Q. 2017;41:74–83.

[3] Thompson JE, Phillips BA, Mccracken A, Thomas K, Ward WL. Social anxiety in obese youth in treatment setting. Child Adolesc Soc Work J. 2013;30:37–47.

[4] Sackl-Pammer P, Özlü-Erkilic Z, Jahn R, Karwautz A, Pollak E, Ohmann S, et al. Somatic complaints in children and adolescents with social anxiety disorder. Neuropsychiatrie. 2018;32:187–195. 30218392 10.1007/s40211-018-0288-8 PMC6290697

[5] Bittner A, Egger HL, Erkanli A, Jane Costello E, Foley DL, Angold A. What do childhood anxiety disorders predict? J Child Psychol Psychiatry. 2007;48(12):1174–1183. 18093022 10.1111/j.1469-7610.2007.01812.x

[6] Pine DS, Cohen P, Gurley D, Brook J, Ma Y. The risk for early-adulthood anxiety and depressive disorders in adolescents with anxiety and depressive disorders. 2017;55:56–64. 10.1001/archpsyc.55.1.56 9435761

[7] Stein DJ, Scott KM, de Jonge P, Kessler RC. Epidemiology of anxiety disorders: from surveys to nosology and back. Dialogues Clin Neurosci. 2017;19(2):127–36. 28867937 10.31887/DCNS.2017.19.2/dstein PMC5573557

[8] Dalrymple KL, Herbert JD, Gaudiano BA. Onset of illness and developmental factors in social anxiety disorder: preliminary findings from a retrospective interview. J Psychopathol Behav Assess. 2007;29(2):101–110.

[9] Dalrymple KL, Zimmerman M. Age of onset of social anxiety disorder in depressed outpatients. J Anxiety Disord. 2011;25(1):131–137. 20832989 10.1016/j.janxdis.2010.08.012 PMC3006477

[10] DeWit DJ, Ogborne A, Offord DR, MacDonald K. Antecedents of the risk of recovery from DSM-III-R social phobia. Psychol Med. 1999;29(3):569–582. 10405078 10.1017/s0033291799008399

[11] Olofsdotter S, Åslund C, Furmark T, Comasco E, Nilsson KW. Differential susceptibility effects of oxytocin gene (OXT) polymorphisms and perceived parenting on social anxiety among adolescents. Dev Psychopathol. 2018;30(2):449–459. doi: 10.1017/S0954579417000967. 28606214

[12] Waite P, Whittington L, Creswell C. Parent–child interactions and adolescent anxiety: a systematic review. Psychopathol Rev. 2014;1:51–76.

[13] Oppenheimer CW, Ladouceur CD, Waller JM, Ryan ND, Allen B, Sheeber L, et al. Emotion socialization in anxious youth: parenting buffers emotional reactivity to peer negative events. J Abnorm Child Psychol. 2017;44(7):1267–1278. 10.1007/s10802-015-0125-5 PMC4955624 26783026

[14] Bynion TM, Blumenthal H, Bilsky SA, Cloutier RM, Leen-Feldner EW. Dimensions of parenting among mothers and fathers in relation to social anxiety among female adolescents. J Adolesc. 2017;60:11–15. 28738315 10.1016/j.adolescence.2017.07.004

[15] Bandelow B, Torrente AC, Wedekind D, Broocks A, Hajak G, Rüther E. Early traumatic life events, parental rearing styles, family history of mental disorders, and birth risk factors in patients with social anxiety disorder. Eur Arch Psychiatry Clin Neurosci. 2004;254(6):397–405. 15538600 10.1007/s00406-004-0521-2

[16] Lewis-Morrarty E, Degnan KA, Chronis-Tuscano A, Rubin KH, Cheah CSL, Pine DS, et al. Maternal over-control moderates the association between early childhood behavioral inhibition and adolescent social anxiety symptoms. J Abnorm Child Psychol. 2012;40:1363–1373. 22821448 10.1007/s10802-012-9663-2 PMC7384351

[17] Gulley L, Oppenheimer C, Hankin B. Associations among negative parenting, attention bias to anger, and social anxiety among youth. Dev Psychol. 2015;50(2):577–585. 10.1037/a0033624 PMC4086836 23815705

[18] Belsky J, Pluess M. Beyond diathesis stress: differential susceptibility to environmental influences. Psychol Bull. 2009;135:885–908. 19883141 10.1037/a0017376

[19] Lipscomb ST, Becker DR, Laurent H, Neiderhiser JM, Shaw DS, Natsuaki MN, et al. Examining morning HPA axis activity as a moderator of hostile, over-reactive parenting on children’s skills for success in school. Infant Child Dev. 2018;27(4):e2083. doi: 10.1002/icd.2083. 30147452 PMC6107075

[20] Burt SA. Rethinking environmental contributions to child and adolescent psychopathology: a meta-analysis of shared environmental influences. Psychol Bull. 2009;135(4):608–637. 19586164 10.1037/a0015702

[21] Moreno AL, De Lima Osório F, Martín-Santos R, Crippa JAS. Heritability of social anxiety disorder: a systematic review of methodological designs. Rev Psiquiatr Clín. 2016;43(4):83–92.

[22] Trzaskowski M, Eley TC, Davis OSP, Doherty SJ, Hanscombe KB, Meaburn EL, et al. First genome-wide association study on anxiety-related behaviours in childhood. PLoS One. 2013;8(4):1–7. 10.1371/journal.pone.0058676 PMC3614558 23565138

[23] Stein MB, Chen CY, Jain S, Jensen KP, He F, Heeringa SG, et al. Genetic risk variants for social anxiety. Am J Med Genet B Neuropsychiatr Genet. 2017;174(2):120–131. 28224735 10.1002/ajmg.b.32520 PMC5325045

[24] Hill WG, Goddard ME, Visscher PM. Data and theory point to mainly additive genetic variance for complex traits. PLoS Genet. 2008;4(2):e1000008. 18454194 10.1371/journal.pgen.1000008 PMC2265475

[25] Hill WG, Mulder HA. Genetic analysis of environmental variation. Genet Res. 2010;92(5–6):381–395. 10.1017/S0016672310000546 21429270

[26] Rosenman R, Tennekoon V, Hill LG. Measuring bias in self-reported data. Int J Behav Healthc Res. 2011;2(4):320–332. doi: 10.1504/IJBHR.2011.043414. 25383095 PMC4224297

[27] Gauderman WJ, Mukherjee B, Aschard H, Hsu L, Lewinger JP, Patel CJ, et al. Update on the state of the science for analytical methods for gene–environment interactions. Am J Epidemiol. 2017;186(7):762–770. 28978192 10.1093/aje/kwx228 PMC5859988

[28] Sullivan PF. Spurious genetic associations. Biol Psychiatry. 2007;61(10):1121–1126. 17346679 10.1016/j.biopsych.2006.11.010

[29] Keller MC. Gene–environment interaction studies have not properly controlled for potential confounders: the problem and the (simple) solution. Biol Psychiatry. 2014;75:18–24. 24135711 10.1016/j.biopsych.2013.09.006 PMC3859520

[30] Franceschini A, Szklarczyk D, Frankild S, Kuhn M, Simonovic M, Roth A, et al. STRING v9.1: protein–protein interaction networks, with increased coverage and integration. Nucleic Acids Res. 2013;41(D1):808–815. 10.1093/nar/gks1094 PMC3531103 23203871

[31] Lee I, Blom UM, Wang PI, Shim JE, Marcotte EM. Prioritizing candidate disease genes by network-based boosting of genome-wide association data. Genome Res. 2011;21:1109–1121. 21536720 10.1101/gr.118992.110 PMC3129253

[32] Schneider-Hassloff H, Straube B, Jansen A, Nuscheler B, Wemken G, Witt SH, et al. Oxytocin receptor polymorphism and childhood social experiences shape adult personality, brain structure and neural correlates of mentalizing. NeuroImage. 2016;134:671–684. doi: 10.1016/j.neuroimage.2016.04.009. 27109357

[33] Van Heel M, Bijttebier P, Claes S, Colpin H, Goossens L, Van Den Noortgate W, et al. Measuring parenting throughout adolescence: measurement invariance across informants, mean level, and differential continuity. Assessment. 2019;26:111–124. doi: 10.1177/1073191116686827. 28076976

[34] Van Assche E, Moons T, Cinar O, Viechtbauer W, Oldehinkel AJ, Van Leeuwen K, et al. Gene-based interaction analysis shows GABAergic genes interacting with parenting in adolescent depressive symptoms. J Child Psychol Psychiatry. 2017;58(12):1301–1309. 28660714 10.1111/jcpp.12766

[35] Brown MB. A method for combining non-independent. One-sided tests of significance. Biometrics. 1975;31:987.

[36] Nelemans SA, Meeus WHJ, Branje SJT, Van Leeuwen K, Colpin H, Verschueren K, et al. Social Anxiety Scale for Adolescents (SAS-A) Short Form: longitudinal measurement invariance in two community samples of youth. Assessment. 2017;26:235–248. 28052690 10.1177/1073191116685808

[37] La Greca, A. M., & Lopez, N. (1998). Social Anxiety Amon Adolescents : Linkages with Peer Relation s and Friendships. Journal of Abnormal Child Psychology,26(2), 83–94. 10.1023/A:1022684520514 9634131

[38] Delhaye M, Beyers W, Klimstra TA, Linkowski P, Goossens L. The Leuven Adolescent Perceived Parenting Scale (LAPPS): reliability and validity with French-speaking adolescents in Belgium. Psychol Belg. 2012;52:289–305.

[39] Wirtz PH, von Känel R. Psychological stress, inflammation, and coronary heart disease. Curr Cardiol Rep. 2017;19(11):111. 28932967 10.1007/s11886-017-0919-x

[40] Van Leeuwen K, Vermulst A, Kroes G, De Meyer R, Nguyen L, Veerman JW. Verkorte Schaal voor Ouderlijk Gedrag (VSOG): Handleiding [Brief Scale of Parental Behavior]. Nijmegen, The Netherlands: Praktikon; 2013.

[41] Janssens A, Goossens L, Van Den Noortgate W, Colpin H, Verschueren K, Van Leeuwen K. Parents’ and adolescents’ perspectives on parenting: evaluating conceptual structure, measurement invariance, and criterion validity. Assessment. 2015;22(4):473–489. 25225229 10.1177/1073191114550477

[42] Anderson CA, Pettersson FH, Clarke GM, Cardon LR, Morris AP, Zondervan KT. Data quality control in genetic case–control association studies. Nat Protoc. 2010;5:1564–1573. 21085122 10.1038/nprot.2010.116 PMC3025522

[43] Rutter M. Gene–environment interdependence. Eur J Dev Psychol. 2012;9(4):391–412.

[44] Liu YZ, Wang YX, Jiang CL. Inflammation: the common pathway of stress-related diseases. Front Hum Neurosci. 2017;11:1–11. 28676747 10.3389/fnhum.2017.00316 PMC5476783

[45] Purcell SM, Wray NR, Stone JL, Visscher PM, O’Donovan MC, Sullivan PF, et al. Common polygenic variation contributes to risk of schizophrenia and bipolar disorder. Nature. 2009;460:748–752. 19571811 10.1038/nature08185 PMC3912837

[46] Legendre P, Legendre L. Statistical testing by permutation In: Legendre P, Legendre L, editors. Numerical ecology. Volume 2, 1998; p. 17–26.

[47] Devlin B, Roeder K. Genomic control for association studies. Biometrics. 1999;55(4):997–1004. 11315092 10.1111/j.0006-341x.1999.00997.x

[48] RStudio Team. RStudio: integrated development for R. Boston, MA: RStudio, Inc., 2017 http://www.rstudio.com/.

[49] Simon NM, Herlands NN, Marks EH, Mancini C, Letamendi A, Li Z, et al. Childhood maltreatment linked to greater symptom severity and poorer quality of life and function in social anxiety disorder. Depress Anxiety. 2009;26(11):1027–1032. 19750554 10.1002/da.20604 PMC2991116

[50] Hovens JGFM, Giltay EJ, Van Hemert AM, Penninx BWJH. Childhood maltreatment and the course of depressive and anxiety disorders: the contribution of personality characteristics. Depress Anxiety. 2016;33(1):27–34. 26418232 10.1002/da.22429

[51] Arnold PD, Macmaster FP, Hanna GL, et al. Glutamate system genes associated with ventral prefrontal and thalamic volume in pediatric obsessive–compulsive disorder. Brain Imaging Behav. 2009;3(1):64–76. 21031159 10.1007/s11682-008-9050-3 PMC2964163

[52] Dickel DE, Veenstra-VanderWeele J, Cox NJ, Wu X, Fischer DJ, Van Etten-Lee M, et al. Association testing of the positional and functional candidate gene SLC1A1/EAAC1 in early-onset obsessive–compulsive disorder. Arch Gen Psychiatry. 2006;63:778–785. 16818867 10.1001/archpsyc.63.7.778

[53] Wu H, Wang X, Yu S, Wang D, Chen J, Jiang K, et al. Association of the candidate gene SLC1A1 and obsessive–compulsive disorder in Han Chinese Population. Psychiatry Res. 2013;209(3):737–739. 23411042 10.1016/j.psychres.2012.12.016

[54] Zhang J, Sheerin C, Mandel H, Banducci AN, Myrick H, Acierno R, et al. Variation in SLC1A1 is related to combat-related posttraumatic stress disorder. J Anxiety Disord. 2014;28(8):902–907. 25445080 10.1016/j.janxdis.2014.09.013

[55] Gadow KD, Roohi J, Devincent CJ, Kirsch S, Hatchwell E. Brief report: glutamate transporter gene (SLC1A1) single nucleotide polymorphism (rs301430) and repetitive behaviors and anxiety in children with autism spectrum disorder. J Autism Dev Disord. 2010;40(9):1139–1145. 20155310 10.1007/s10803-010-0961-7 PMC4348063

[56] Blackburn AC, Woollatt E, Sutherland GR, Board PG. Characterization and chromosome location of the gene GSTZ1 encoding the human Zeta class glutathione transferase and maleylacetoacetate isomerase. Cytogenet Cell Genet. 1998;83(1–2):109–114. 9925947 10.1159/000015145

[57] Board PG, Menon D. Glutathione transferases, regulators of cellular metabolism and physiology. Biochim Biophys Acta. 2013;1830(5):3267–3288. 23201197 10.1016/j.bbagen.2012.11.019

[58] Rezaei Z, Saadat I, Saadat M. Association between three genetic polymorphisms of glutathione S-transferase Z1 (GSTZ1) and susceptibility to bipolar disorder. Psychiatry Res. 2012;198(1):166–168. 22374552 10.1016/j.psychres.2011.09.002

[59] Shao Y, Yan G, Xuan Y, Peng H, Huang Q. Chronic social isolation decreases glutamate and glutamine levels and induces oxidative stress in the rat hippocampus. Behav Brain Res. 2015;282:201–208. 25591473 10.1016/j.bbr.2015.01.005

[60] Hovatta I, Barlow C. Molecular genetics of anxiety in mice and men. Ann Med. 2008;40(2):92–109. 18293140 10.1080/07853890701747096

[61] Modi S, Rana P, Kaur P, Rani N, Khushu S. Glutamate level in anterior cingulate predicts anxiety in healthy humans: a magnetic resonance spectroscopy study. Psychiatry Res. 2014;224(1):34–41. 25156662 10.1016/j.pscychresns.2014.03.001

[62] Zwanzger P, Zavorotnyy M, Gencheva E, Diemer J, Kugel H, Heindel W, et al. Acute shift in glutamate concentrations following experimentally induced panic with cholecystokinin tetrapeptide—a 3T-MRS study in healthy subjects. Neuropsychopharmacology. 2013;38(9):1648–1654. 23463151 10.1038/npp.2013.61 PMC3717541

[63] Kusano S, Kukimoto-Niino M, Hino N, Ohsawa N, Okuda K, Sakamoto K, et al. Structural basis for extracellular interactions between calcitonin receptor-like receptor and receptor activity-modifying protein 2 for adrenomedullin-specific binding. Protein Sci. 2012;21(2):199–210. 22102369 10.1002/pro.2003 PMC3324764

[64] Aiyar N, Rand K, Elshourbagy NA, Zeng Z, Adamou JE, Bergsma DJ, et al. A cDNA encoding the calcitonin gene-related peptide type 1 receptor. J Biol Chem. 1996;271(19):11325–11329. 8626685 10.1074/jbc.271.19.11325

[65] Pozsgai, G., Liang, L., & Brain, S. D. (2010). Vascular Actions of CGRP and Adrenomedullin: Mechanisms and Potential Contribution to Inflammation in the Cutaneous Microvasculature In D. L. Hay & I. M. Dickerson (Eds.), The calcitonin gene-related peptide family: form, function and future perspectives (pp. 115–130). Dordrecht: Springer Netherlands 10.1007/978-90-481-2909-6_8

[66] Karsan N, Goadsby PJ. Calcitonin gene-related peptide and migraine. Curr Opin Neurol. 2015;28(3):250–254. 25887765 10.1097/WCO.0000000000000191

[67] Wolf YI, Grishin NV, Koonin EV. Estimating the number of protein folds and families from complete genome data. J Mol Biol. 2000;299:897–905. 10843846 10.1006/jmbi.2000.3786

