# Supplementary material for: Gene–environment interaction: New insights into perceived parenting and social anxiety among adolescents
Source: Eur Psychiatry. 2020 Jun 8;63(1):e64. doi: 10.1192/j.eurpsy.2020.62 (PMC7355173; doi:10.1192/j.eurpsy.2020.62)

Supplementary materials

1. Interactions plots

2. The list of the top genes for gene-based G×E interactions (adjusted Brown's method) for five parenting dimensions.

1.

Interaction effects of the most significant SNPs with harsh parental control in predicting social anxiety in adolescents (A) rs474088, *SLC1A1* gene; (B) rs1468951, *GSTZ1* gene; (C) rs12621307, *CALCRL* gene.

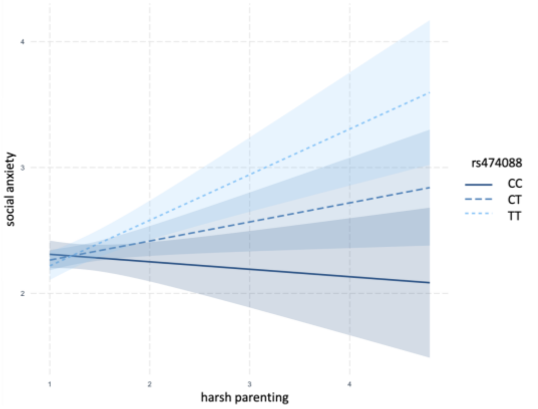

(A)

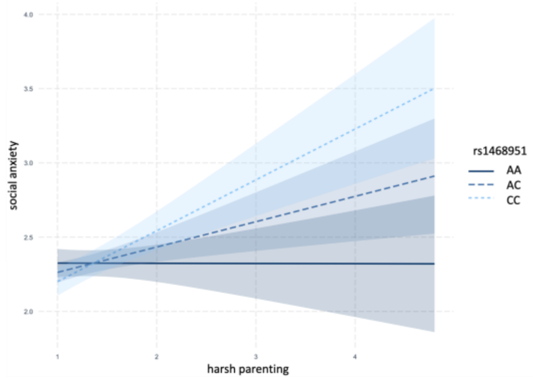

(B)

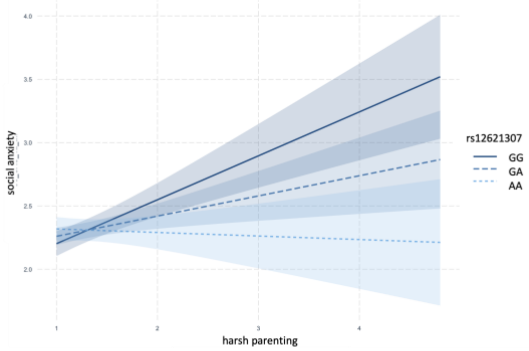

(C)

2.

The list of the top genes for gene-based G×E interactions (adjusted Brown's method) for five parenting dimensions (*harsh punitive control, support, proactive control, psychological control and punitive control*) and Manhattan plots for gene-based G×E interactions (each line represents a gene).

Table 2. The most significant genes for gene-based G×E interactions for Harsh punitive control

Gene	p.value per gene
SLC1A1	**9.35E-05**
GSTZ1	**9.35E-05**
SP1	0.0003
EXOC3	0.0005
ANKK1	0.0005
PER2	0.0005
KCNJ6	0.0006
SLC1A3	0.001
RELN	0.002
CALCRL	0.003
GABRA5	0.003
GRIN3A	0.005
DRD2	0.006

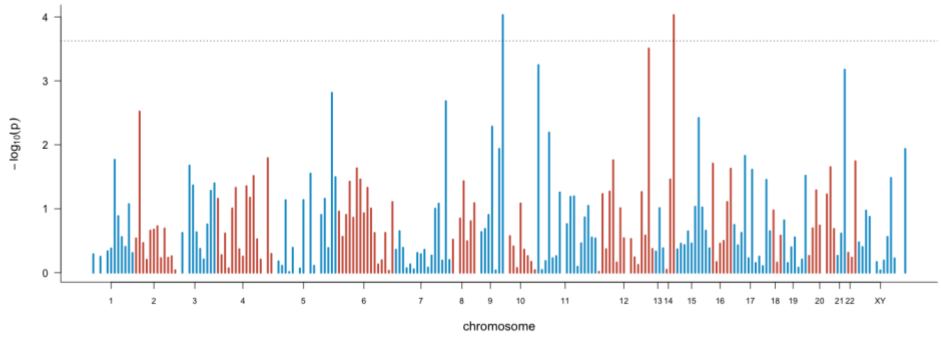

Table 3. The most significant genes for gene-based G×E interactions for Support

Gene	p.value per gene
FAH	0.0007
RELN	0.004
NOS3	0.009
ANKK1	0.017
BHLHE40	0.026
TCF20	0.026
GRIA4	0.027
CHAT	0.028
GHSR	0.029
SNPH	0.032
PER2	0.037
GSTZ1	0.038
NSF	0.038

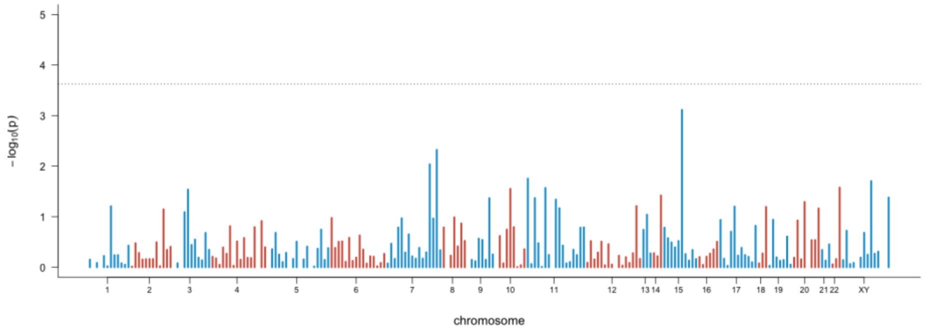

Table 4. The most significant genes for gene-based G×E interactions for Proactive control

Gene	p.value per gene
GRIK3	0.0005
GABRD	0.001
GRM4	0.001
CAMK2A	0.001
CMTM8	0.001
NPY	0.003
DBP	0.004
DAO	0.008
POMC	0.008
SLC1A3	0.019
CRHR1	0.028
HTR7	0.028
SHANK1	0.031
VAMP4	0.04

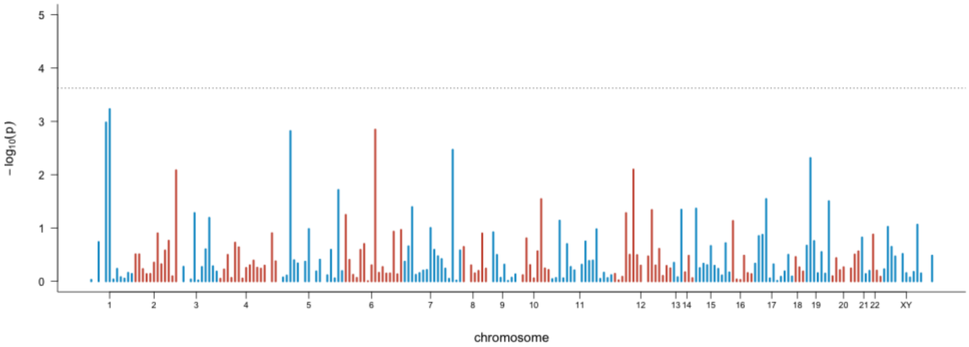

Table 5. The most significant genes for gene-based G×E interactions for Psychological control

Gene	p.value per gene
GABRA5	0.003
HTR3B	0.004
GHRL	0.005
BHLHE41	0.01
GSTZ1	0.01
GRIN1	0.01
VAPA	0.01
RELN	0.01
MC2R	0.01
NOS3	0.01
HTR3A	0.02
ADRB1	0.02
CHRM2	0.03
EXOC3	0.03

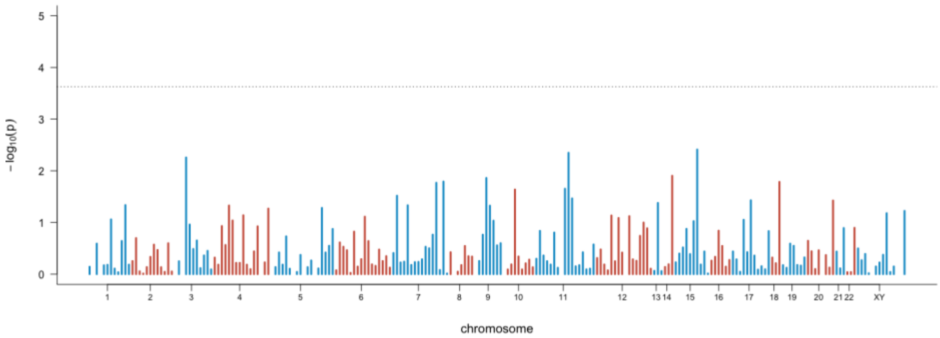

Table 6. The most significant genes for gene-based G×E interactions for Punitive control

Gene	p.value per gene
FAAH	0.0003
GRM4	0.005
AFAP1L1	0.006
HTR1D	0.006
SLC1A1	0.009
CHRNB1	0.01
ARNT2	0.01
PAWR	0.01
SHANK1	0.01
GAD2	0.02
NCAM1	0.02
GIT1	0.02
GRIK3	0.03
MC1R	0.03

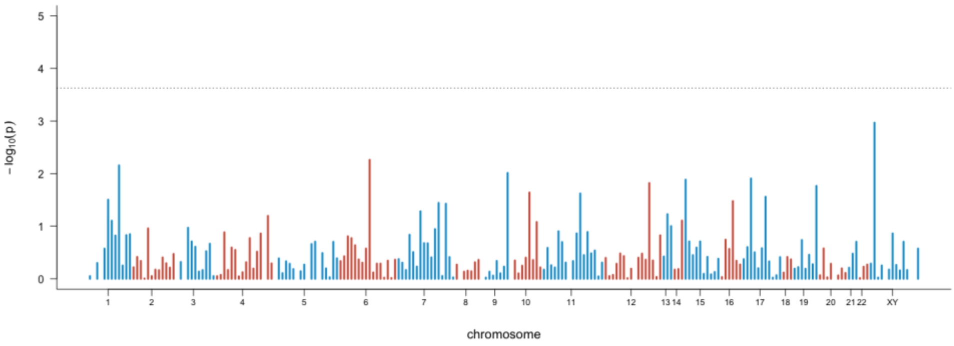

Sensitivity analysis

Table 7. The most significant genes for gene-based G×E interactions for binary Harsh punitive control

Gene	p.value per gene
CALCRL	**1.59E-05**
VAMP3	0.003
ANK3	0.005
GABRA4	0.007
SP1	0.008
GRM4	0.009
CLOCK	0.009
NCAM1	0.01
GPX1	0.01
EXOC3	0.01
GABRB1	0.01
CHRNA2	0.01
CRHR1	0.02
SLC1A3	0.02
GABRA5	0.02

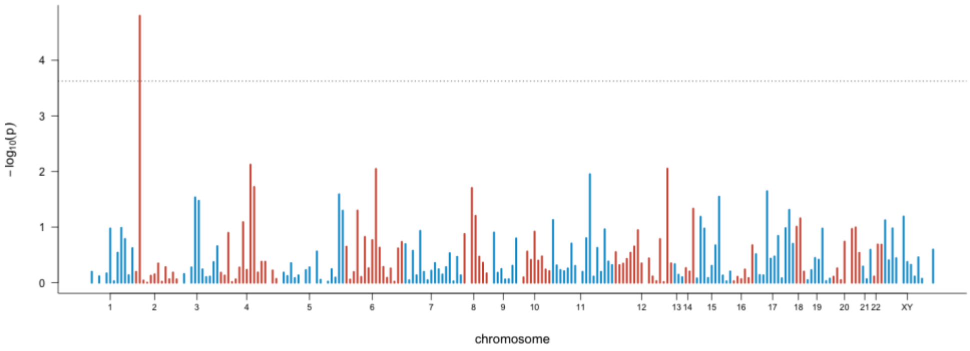

Supplement: Supplementary file 1 [file S0924933820000620sup001.pdf]
